# Tumour Necrosis Factor-alpha and Nuclear Factor-kappa B Gene Variants in Sepsis

**DOI:** 10.4274/balkanmedj.2017.0246

**Published:** 2018-01-20

**Authors:** Leyla Acar, Nazan Atalan, E. Hande Karagedik, Arzu Ergen

**Affiliations:** 1 Department of Molecular Medicine, İstanbul University Institute of Experimental Medicine, İstanbul, Turkey; 2 Clinic of Anesthesia and Reanimation, Siyami Ersek Thoracic Cardiovascular Surgery Training and Research Hospital, İstanbul, Turkey

**Keywords:** Sepsis, polymorphism, tumor necrosis factor-alpha, NF-kappa B

## Abstract

**Background:**

The humoral system is activated and various cytokines are released due to infections in tissues and traumatic damage. Nuclear factor-kappa B dimers are encoded by nuclear factor-kappa B genes and regulate transcription of several crucial proteins of inflammation such as tumour necrosis factor-alpha.

**Aims:**

To investigate the possible effect of polymorphisms on tumour necrosis factor-alpha serum levels with clinical and prognostic parameters of sepsis by determining the nuclear factor-kappa B-1-94 ins/del ATTG and tumour necrosis factor-alpha (-308 G/A) gene polymorphisms and tumour necrosis factor-alpha serum levels.

**Study Design:**

Case-control study.

**Methods:**

Seventy-two patients with sepsis and 104 healthy controls were included in the study. In order to determine the polymorphisms of nuclear factor-kappa B-1-94 ins/del ATTG and tumour necrosis factor-alpha (-308 G/A), polymerase chain reaction–restriction fragment length polymorphism analysis was performed and serum tumour necrosis factor-alpha levels were determined using an enzyme-linked immunosorbent assay.

**Results:**

We observed no significant differences in tumour necrosis factor-alpha serum levels between the study groups. In the patient group, an increase in the tumour necrosis factor-alpha serum levels in patients carrying the tumour necrosis factor-alpha (-308 G/A) A allele compared to those without the A allele was found to be statistically significant. Additionally, an increase in the tumour necrosis factor-alpha serum levels in patients carrying tumour necrosis factor-alpha (-308 G/A) AA genotype compared with patients carrying the AG or GG genotypes was statistically significant. No significant differences were found in these 2 polymorphisms between the patient and control groups (p>0.05).

**Conclusion:**

Our results showed the AA genotype and the A allele of the tumour necrosis factor-alpha (-308 G/A) polymorphism may be used as a predictor of elevated tumour necrosis factor-alpha levels in patients with sepsis.

Sepsis is a multisystem disease typically causing hemodynamic alterations, shock, organ dysfunction, and organ failure. Moreover, sepsis can be life threatening. Studies have shown that the humoral system is activated and various cytokines are released due to infections in tissues and traumatic damage (1). Nuclear factor-kappa B (NF-κB) dimers, which are encoded by NF-κB genes, regulate transcription of several crucial proteins of inflammation such as tumour necrosis factor-alpha (TNF-α) (2). Additionally, NF-κB is a key molecule in the immune and inflammatory response; in many cell types it modulates cell proliferation, apoptosis, adhesion, invasion and angiogenesis (3). The major form of NF-κB is a heterodimer of the p50 and p65/RelA subunits encoded by genes NF-κB1 and RelA (4). -94 ins/del ATTG rs28362491 polymorphism was identified between 2 main promoter regulatory elements in the NF-κB1 gene. ATTG deletion leads to the loss of binding to nuclear proteins, which causes decreased promoter activity (2). ATTG deletion leads to the loss of binding to nuclear proteins, which causes decreased promoter activity. Studies report that the ATTG deletion is associated with immune and inflammatory diseases (2). Schäfer et al. (5) suggested that hydrocortisone therapy in D allele carriers of the NF-κB1-94 ins/del ATTG polymorphism was a prognostic factor for 30-day mortality. TNF-α is one of the major mediators of the immune response against infectious challenge. Increased levels of TNF-α have been found in sepsis, but TNF-α inhibition has proven to be an unsuccessful treatment (6). The main source of TNF-α synthesis is human monocytes. Plasma levels of TNF-α are normally minimal; however, cells of the immune system release TNF-α on stimulation. The correlation between TNF-α and inflammatory process-based pathologic states has been previously investigated. In healthy volunteers and patients with septic shock, elevated TNF-α plasma levels have been reported after endotoxin excitation (7). Several polymorphisms have been identified at different positions of the TNF-α promoter region in various infectious and inflammatory diseases (8,9), mainly focusing on the promoter single nucleotide polymorphism TNF-α-308 G/A in sepsis. Although many studies have reported the association of the A allele with susceptibility to septic shock, the findings have been variable (10,11). The G/A transition at position -308 in the TNF-α promoter has been reported to affect TNF promoter activity and TNF-α production (12). In a meta-analysis, Zhang et al. (13) reported that TNF-α-308 G/A and -238 G/A polymorphisms were associated with an increased risk of sepsis but not sepsis-related mortality.

The aim of this study was investigate the possible effects of NF-κB1 -94 ins/del ATTG and TNF-α (-308 G/A) gene polymorphisms on TNF-α serum levels and clinical parameters of sepsis in Turkish sepsis patients.

## MATERIALS AND METHODS

### Study groups

The study group consisted of 72 patients (21 women and 51 men; mean age 64.60±11.45 years) diagnosed with sepsis in between February 2014 and February 2015. Sepsis was defined in accordance with Bone et al. (14). The control group included 104 randomly selected healthy individuals (20 women, 84 men; mean age 62.28±11.34 years) who had no signs of sepsis. All participants gave written informed consent. This study was approved by the local ethics committee (February 20, 2014; number: 380) and was carried out in concordance with the Declaration of Helsinki.

### DNA isolation

Peripheral blood of participants was collected in EDTA-containing vacurtainer tubes (BD Vacutainer, Franklin Lakes, NJ., USA) Genomic DNA was extracted in accordance with PureLink® Genomic Kit manufacturer's instructions (Invitrogen, Carlsbad, CA, USA).

### Genotyping for NF-κB1-94 ins/del ATTG and TNFα (-308 G/A) polymorphisms

The NF-κB1 -94 ins/del ATTG and TNF-α-308G/A polymorphisms were amplified and genotyped using polymerase chain reaction–restriction fragment length polymorphism (PCR-RFLP). The genotype of the *NF-κB1* gene polymorphism was amplified using the following primer sequences: F- 5'-TTT AAT CTG TGA AGA GAT GTG AAT G -3', R- 5'- CTC TGG CTT CCT AGC AGG G -3' and the genotype of the TNF-α-308 G/A gene polymorphism was amplified using the following primer sequences: F- 5'-AGG CAA TAG GTT TTG AGG GCC AT -3', R- 5'- TCC TCC CTG CTC CGA TTC CG -3'.

PCR was performed for the *NF-κB1* gene with the initial denaturation at 95 ºC for 5 minutes, 38 cycles at 94 ºC for 45 seconds, 72 ºC for 45 seconds and the final step at 72 ºC for 5 minutes. PCR products were then digested using the Van91I restriction enzyme for 3 hours at 37 ºC. Three genotypes were determined through distinct banding patterns as base pairs (bp) on a 2% agarose gel for the *NF-κB1* gene polymorphism: homozygous del/del (254 bp), heterozygous ins/del (254 and 206 bp) and homozygous ins/ins (206 bp).

For the TNF-α-308 G/A polymorphism, PCR was performed with the initial denaturation at 95 ºC for 5 minutes, 35 cycles of 94 ºC for 45 seconds, 64 ºC for 45 seconds, 72 ºC for 45 seconds and the final step at 72 ºC for 5 minutes. The PCR products were digested using the NcoI restriction enzyme for 3 hours at 37 ºC. Three genotypes were determined through distinct banding patterns on a 2% agarose gel for the TNF-α gene polymorphism: 107 bp for the AA genotype, 20 and 87 bp for the GG genotype, and 20, 87 and 107 bp for the AG genotype.

### Determination of TNFα levels

Fasting blood samples were obtained from each participant in plain tubes (Vacuette). The samples were centrifuged for 10 minutes at 1500 × g followed by the removal of serum. Levels of TNF-α were determined using a human ELISA kit (Diaclone, Besancon Cedex, France) in Greiner Labortechnik, Germany, accordance with the manufacturer's protocol.

### Statistical analysis

The statistical analyses were performed using the SPSS software version 21.0 (SPSS, Chicago, Illinois, USA). P values less than 0.05 were assumed to be statistically significant. The differences in allele and genotype frequencies between patient and control groups were detected using the χ2 and Fisher test. We compared the biochemical parameters in the case and the control groups using the Student’s t-test. Biochemical parameters among the genotypes were investigated using One-Way ANOVA and the Mann-Whitney U test.

## RESULTS

### Biochemical and demographical analysis

Biochemical and demographic data of the study groups are given in Table 1. No statistical difference was found in terms of age between the patient and the control groups (p>0.05). As expected, body temperature, creatinine, blood urea nitrogen (BUN), white blood cells (WBC), C-reactive protein (CRP), pH, lactate, glucose, lactate dehydrogenase, serum glutamic-oxaloacetic transaminase (SGOT), serum glutamic-pyruvic transaminase (SGPT) (p<0.001) and K+ (p=0.043) levels are higher in the sepsis group compared to the controls.

### Genetic analysis

Between the study groups, there were no significant findings in terms of the *NF-κB1* -94 ins/del ATTG and TNF-α (-308 G/A) polymorphism genotypes and allele distributions (Table 2).

### Hardy-Weinberg equilibrium

Each of the case and control groups was checked for all polymorphisms using Hardy-Weinberg equilibrium, and the equilibrium was confirmed by PLINK software using the exact test (for patients *NF-κB1* p=0.006, TNF-α p=0.02; for controls *NF-κB1* p=0.001, TNF-α p=0.38).

Serum TNF-α levels according to *NF-κB1* -94 ins/del ATTG and TNF-α (-308 G/A) genotypes and allele distributions in the study groups are shown in Table 3. In the patient group, increased TNF-α serum levels in patients carrying the TNF-α (-308 G/A) A allele compared with those without the A allele were found to be statistically significant. Additionally, increased TNF-α serum levels in patients carrying TNF-α (-308 G/A) AA genotype was statistically significant when compared to patients carrying the GA genotype [95% CI: (10.71-71.66); p=0.009] and GG [p=0.002, 95% CI: (16.92-72.33)]. When comparing *NF-κB1* -94 ins/del ATTG, TNF-α (-308 G/A) genotype and allele distributions in the control group, no significant difference between TNF-α levels was found. TNF-α levels in terms of NF-kB1 -94 ins/del ATTG polymorphism were determined as ins/ins>ins/del>del/del, and TNFα levels in terms of TNF-α (-308 G/A) polymorphism were determined as AA>GA>GG respectively. We also detected TNF-α levels by infectious agent (Table 4). In patients, TNF-α levels increased in the presence of *Acinetobacter baumanni, Corynebacterum striatum, Streptococcus pneumonia* and *Serratia marcescens *when compared to infectious agents.

### Multivariate logistic regression analysis

Multivariate logistic regression analysis was also performed and no significant results were found.

## DISCUSSION

The common parameters for the diagnosis of sepsis were redefined in 2005. We observed compatible clinical parameters in the patients (15). In the present study, there was a statistically significant increase for the following parameters in the patient group compared to healthy group: creatinine, BUN, WBC, CRP, lactate, fasting blood glucose, K+, SGPT, SGOT, lactate dehydrogenase levels and body temperature. Two initial molecules in sepsis that need further exploration are TNF-α and interleukin-1 (IL-1). These inflammatory factors associated with severe sepsis are shown by some studies (16,17,18).

Single nucleotide polymorphisms such as TNF-α, IL-1, IL10, and *Fc-γ* receptor genes were found to be effective in the regulation of the inflammatory response against microorganisms. Polymorphisms in cytokine genes can affect inflammatory or anti-inflammatory cytokine production and concentrations. Consequently, the patients have either an increased or decreased inflammatory response. It was detected that various genetic polymorphisms, including TNF-α, IL-1, IL-6, and IL- 10, were associated with a predisposition to infection and increased mortality in patients with sepsis (17,18). In a GWAS study in premature infants, Srinivasan et al. (19) did not report any significant results for common sepsis genes, such as IL-6, TLR-2, TLR-4, etc.

Previous studies demonstrated that cytokine gene polymorphisms caused severe pneumococcal and meningococcal infections and septic shock (20,21,22). A Turkish study demonstrated the MyD88 SNP -938 C/C genotype was associated with sepsis (23). Tak and Firestein (22) observed that levels of IL-1β, IL-6, and TNF-α proinflammatory proteins in the cell were reduced by suppression of NF-κB dependent inflammation. Additionally, a positive relationship was observed between increased cellular IL-6 levels and the *NF-κB1* -94 ins/del ATTG polymorphism deletion allele in a study by Giachelia et al. (24).

In their study comparing carriers of the -308 A allele with GG homozygous individuals in patients with burns, Barber et al. (25) determined that the risk of developing severe sepsis was higher in -308A allele carriers. Song et al. (26) suggested that -308 A was strongly associated with susceptibility to severe sepsis, but not with mortality in the Chinese Han population. Feng et al. (27) reported that TNF-α-308 A allele and the IL-6 rs1800795 allele variants were risk factors for septic shock induced by pneumonia in intensive care unit patients. Baghel et al. (28) indicated that the TNF-α-308 G/A polymorphism was associated with the development of postoperative sepsis and increased expression of TNF-α, IL-6 and IL-8 genes. Contrary to these studies, we did not find any significant differences between patients and controls according to TNF-α genotype and allele distributions.

In 1993, de Bont et al. (29) reported that in neonatal sepsis IL-6 and TNF-α serum levels increased significantly but IL-1β increased only slightly. In addition, in 1994, Ozdemir et al. (30) detected that IL-1β also increased in neonatal sepsis. In 1993, Casey et al. (31) concluded that patients carrying sepsis syndrome criteria, TNF-α, IL-1β and IL-6 levels were high, and there was an inverse correlation between high IL-6 and survival regardless of microbiologic profiles. In 1994, van Deuren et al. (32) specified that in fulminant septicemia, TNF-α levels increased briefly and temporarily for a time in the early stages of infection, but the production of IL-1β was never induced. Kothari et al. (33) reported that plasma TNF-α levels and the single nucleotide polymorphism of the *TNF* gene showed significant association with the development of severe sepsis and septic shock. Bavunoglu et al. (34) suggested that IL-6, TNF-α, NT, and oxLDL serum levels were correlated with the severity of sepsis.

In our study, we compared serum TNF-α levels between the study groups and found increased TNF-α levels statistically insignificant. We suggest that the real importance of TNF-α, which is thought to play an important role in the pathogenesis of sepsis, and the periods in which TNF-α plays an active role during the disease should be determined in large population studies. According to TNF-α genotypes and alleles, we observed that TNF-α serum levels in patients carrying TNF-α (-308 G/A) A allele had statistically significant increases compared to those without the A allele. Additionally, TNF-α serum levels in patients carrying TNF-α (-308 G/A) AA genotype showed a statistically significant increase compared to patients carrying GA.

Karban et al. (2) showed that the promoter activity of the *NF-κB1* -94 del ATTG del allele is considered low, but the promoter activity of the ins allele is considered high. Therefore, the del allele may lead to the production of the p50/p105 NF-κB heterodimer at lower levels and reduce the inflammatory response causing a decrease in *NF-κB1* expression.

The *NF-κB1* -94 ins/del ATTG promoter gene polymorphism was studied and found to also have some immune inflammatory diseases (35). The results obtained from these studies contradict each other as different results were obtained even between experiments that studied the same disease group. These contradictions have been attributed to racial differences (36). Adamzik et al. (37) found that the *NF-κB1* -94 ins/del ATTG del allele is associated with an increased 30-day mortality rate in severe sepsis and an increased activation of the innate immune system. In our study, no statistically significant finding was encountered in terms of the *NF-κB1* -94 ins/del ATTG polymorphism genotype and allele distributions between study groups.

The transcription of TNF-α is performed by NF-κB (3). Therefore, it is expected that increased NF-κB activity can cause an increase in TNF-α expression. However, in our study, no significant difference in TNF-α levels was found.

Our findings suggest that there is no relationship between sepsis risk and both polymorphisms; however, the AA genotype and A allele are associated with increased TNF-α levels in patients. As a result, although there have been many innovations in the genetic knowledge and treatment of sepsis, we think that further studies on polymorphisms will be useful, and the data obtained in this study must be supported by further studies with increased patient participation.

## Figures and Tables

**Table 1:**
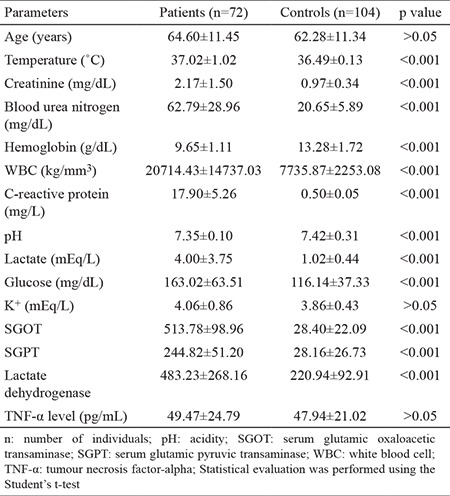
Biochemical and demographic parameters of the study population

**Table 2:**
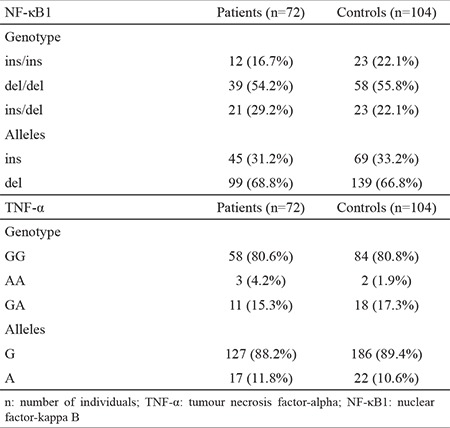
Distribution of NF-κB1 -94 ins/del ATTG and TNF-α (-308 G/A) genotype and alleles in the study groups

**Table 3:**
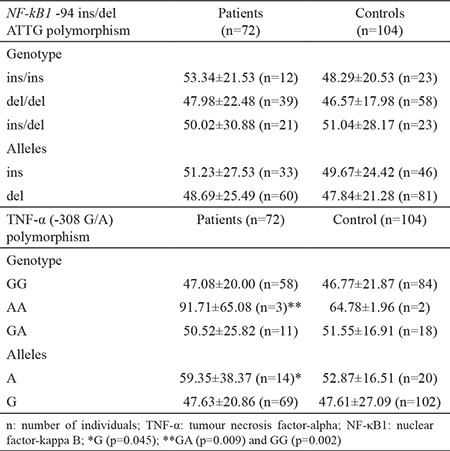
Serum TNF-α levels according to NF-κB1 -94 ins/del ATTG and TNF-α (-308 G/A) genotypes and alleles

**Table 4:**
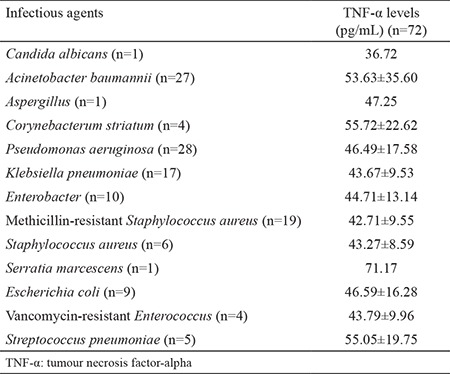
TNF-α levels according to infectious agent in patients
